# Understanding of RF Cloud Interference Measurement and Modeling

**DOI:** 10.1007/s10776-021-00541-8

**Published:** 2021-12-18

**Authors:** Kaveh Pahlavan

**Affiliations:** Worcester Polytechnic Institute, Worcester, MA USA

**Keywords:** RF Cloud, Cyberspace applications, Intelligent spectrum management, Interference modelling, Interference monitoring, Frequency regulations

## Abstract

Importance of spectrum regulation and management was first revealed on May of 1985 after the release of unlicensed ISM bands resulting in emergence of Wi-Fi, Bluetooth and many other wireless technologies that has affected our daily lives by enabling the emergence of the smart world and IoT era. Today, the idea of a liberated spectrum is circulating around, which can potentially direct wireless networking industry into another revolution by enabling a new paradigm in intelligent spectrum regulation and management. The RF signal radiated from IoT devices as well as other wireless technologies create an RF cloud causing co- and cross-interference to each other. Lack of a science and technology for understanding, measurement, and modeling of the RF cloud interference in near real-time results in inefficient utilization of the precious spectrum, a unique natural resource shared among all wireless devices of the universe in frequency, time, and space. Near real time forecasting of the RF cloud interference is essential to pursue the path to the optimal utilization of spectrum and a liberated spectrum management. This paper presents a historical perspective on the evolution of spectrum regulation and management, explains the diversified meanings of interference for different sectors of the wireless industry, and presents a path for implementing a theoretical foundation for interference monitoring and forecasting to enable the emergence of a liberated spectrum industry and a new paradigm in spectrum management and regulations.

## Introduction

In May of 2002 at 50th anniversary of the IEEE Communications Magazine, in the special issue devoted to this event, they reprinted Andy Viterbi’s tutorial “Spread Spectrum Communications: Myths and Realities” [1]. In his commentary on the reprint, Andy wrote: “Until that time (1979), the prominent use of Spread Spectrum was for military anti-jam and secure communication systems and for ranging in military and space missions. Today (2002) there are well over one hundred million consumers using devices that employ spread spectrum technology …..”. He continues by referring to applications of the spread spectrum technology in the global positioning system (GPS) for localization and code division multiple access (CDMA) for the third generation (3G) cellular telephone network to underline the impact of this technology on our daily lives [1]. According to Viterbi this impact is explained by “The creation of GPS by the US Government, …, (that) provided mobile consumer the ability to determine their position practically anywhere on the earth. But the great majority of the growth was the result of the proliferation of wireless cellular ……to serve billions of consumers”. Another mystic application of the spread spectrum technology that emerged in that era was the wireless local area networking [2], what is known as Wi-Fi today, which became the most popular wireless access technology for the Internet protocol (IP) traffic, the enabler of the smart world and the IoT [3].

Today, since GPS works in 99% of the vast outdoor areas except indoors [4], where most popular smart world applications and IoT devices exist, Wi-Fi and cell tower positioning support those indoor applications [5, 6]. In wireless networking, over a billion Wi-Fi access points, millions of 5G cellular base stations, and several billion smart phones carrying 5G cellular, Wi-Fi, and Bluetooth chipsets for communications to enable formation of the IoT to communicate with close to hundred billion IoT devices and construct the smart world. The RF signal radiating from these devices forms an RF cloud enabling emergence of numerous cyberspace applications for positioning [6], human-computer-interfacing, gesture and motion detection, and authentication and security [3, 7]. The RF cloud of each wireless device also contributes co- and cross-channel interference to other wireless devices in its area of coverage in and around its frequency of operation. Measurement and modelling of the interference contents of the RF cloud is essential for intelligent spectrum management and regulation [8].

However, like spread spectrum technology, interference management has its own “Myths and Realities” caused by lack of a clear understanding of the meaning of the differences among intentional interference in military applications and unintentional interference in commerce as well as complexity of interference analysis in a constantly changing scenario of operation for billions of devices sharing the spectrum. Lack of a clear understanding of the meaning of interference for different industries has already created delays in the growth of several wireless communications networking technologies causing inefficiencies in economic growth and threatening US leadership in these areas. A theoretical foundation for interference monitoring and modeling will help this situation. The current spectrum management systems monitor the interference for spectrum management at fixed base stations for spectrum sharing in the C and L bands. With growth of the IoT and emergence of the smart world we need an intelligent interference forecasting system (IIFS) to enable intelligent spectrum management at device level to lead us to a liberated spectrum access environment.

In this paper we provide a holistic view of interference among different sectors of the industry and propose a path for the design of a centralize IIFS for near real-time spectrum monitoring and prediction. We begin with classification of major RF cloud contributors, and a historical overview of interference management and regulations. Then we provide an overview of the economic, political, and industrial meaning of interference for different sectors of the industry affected by interference management and how they can benefit from interference monitoring. Finally, we present a framework to establish a theoretical foundation for scientific understanding of RF cloud interference and propose a practical approach for creation of an IIFS.

## RF Cloud and Interference Regulations and Management

**Fig. 1:**
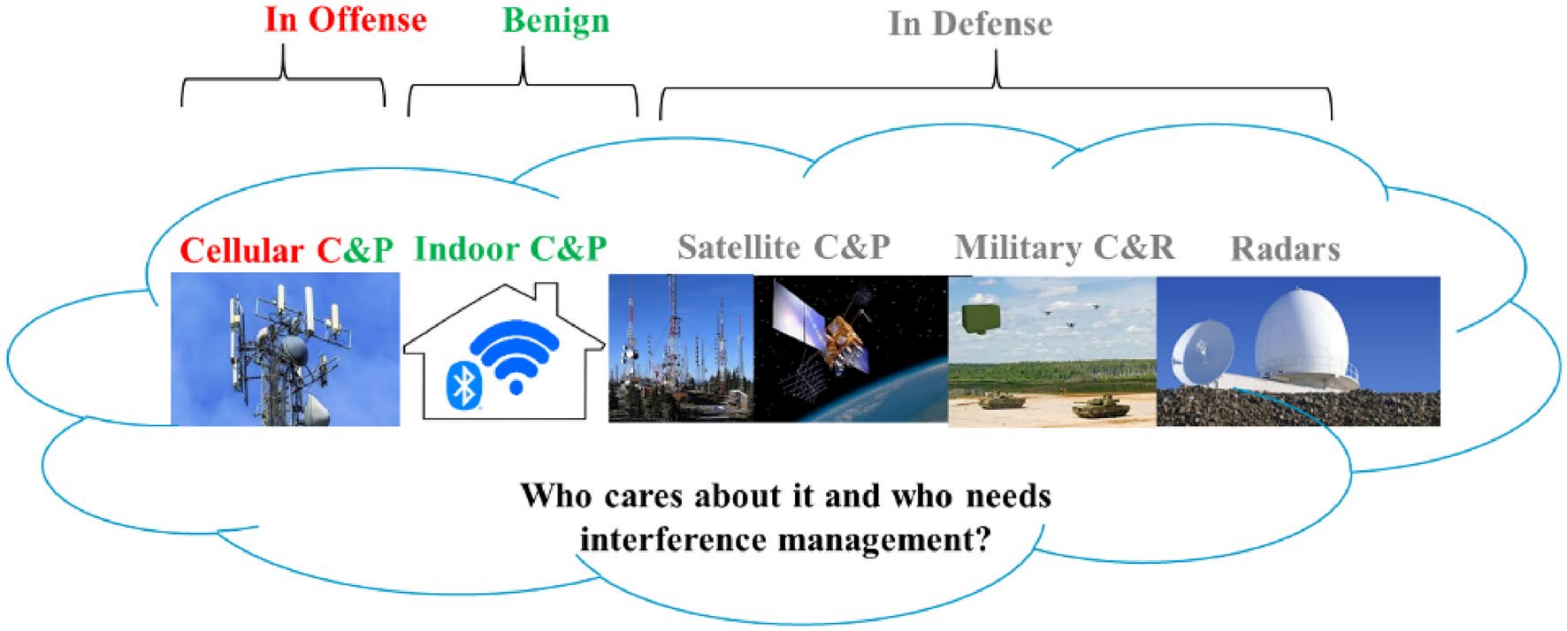
Contributors to RF cloud and the nature of interference

RF cloud is the radio wave propagated from RF devices in space in patterns guided by the devices’ antennas, which range from close to ideal isotropic antennas propagating in all dimensions to massive multiple-input multiple output (MIMO) antenna systems with focused beam widths on the orders of a few degrees [9]. Figure 1 shows the most common contributors to RF cloud for terrestrial and satellite wireless access and localization services in commercial and military applications. We can divide these into wireless communications devices in urban and indoor areas (cellular wireless, Wi-Fi, Bluetooth) [9], positioning systems (GPS, Wi-Fi, cellular) [6], broadcasting services (radio and TV), and radars (astronomy, defense, navigation) [10]. All these contributors to the RF cloud are sharing the same medium for RF propagation, the “air”, and cause co-channel and cross-channel interference to others in their area of coverage demanding government rules and regulations. Coverage of these devices varies from a few meters for RFIDs and short-range radars for motion monitoring up to astronomic distances for satellite communications and astronomy radars. Frequency spectrum is a unique natural resource shared among all these services by multiplexing in time, frequency, or space. In the U.S. Federal Communications Commission (FCC), overseen by the Congress, is responsible for implementation and enforcement of U.S. radio communications laws and regulations to manage the interference in location, frequency, and more recently in time. The most dominant quantitative measure for interference is the received signal to interference ratio (SIR) also referred to as carrier to noise ratio (C/I) and FCC allocates bands according to different models of co- and adjacent-channel SIR levels.

### The Basic Model for SIR

The received signal to noise ratio (SNR) and the signal to noise plus interference ratio (SNIR) are defined as:

1a $$SNR = \frac{{P_{r - D} }}{{N_{0} W}}\,\,\,\,\,and\,\,\,SNIR = \frac{{P_{r - D} }}{{N_{0} W + P_{r - I} }},$$

where *P*_*r-D*_ is the received power from a desired source,_*-*_*P*_*r-I*_ is the received power from an interfering device, *N*_*0*_ is the density of background thermal noise, and *W* is the bandwidth of the receiver. In this formulation SIR is given by:

$$SIR = \frac{{P_{r - D} }}{{P_{r - I} }}.$$

The received power, *P*_*r*_, from a wireless transmitter follow the Friis equation [11, 12] and it is modeled as:

$$P_{r} = GP_{t} \left( {\frac{\lambda }{4\pi d}} \right)^{\alpha } ,$$

where *P*_*t*_* is* the transmitted power, *λ*, is the wavelength of the carrier frequency, *G* is the antenna gain (function of angular selectivity of the antennas), α is the distance-power gradient of the environment, and *d* is the distance between the transmitter and the receiver. Therefore, assuming normalized antenna gains, the relation between SIR and transmitted power from the desired and an interfering device is:

1b $$SIR = \frac{{P_{r - D} }}{{P_{r - I} }} = \frac{{P_{t - D} }}{{P_{t - I} }}\left( \frac{D}{R} \right)^{\alpha } \, ,$$

where *R and D,* are distances between the desired and interfering sources from a target receiver, respectively. In practice, wireless access and localization devices are designed with a spectrum mask that ensures their proper performance inside the allocated spectrum and a controlled cross-channel interference with other wireless device operating in the neighboring bands. The co-channel interference becomes important during the geographical deployment of antennas for certain services to manage frequency-reuse and interference. In finding the geographical location for a broadcast radio or TV antenna tower, in planning for cellular deployment of a wireless network, or in installing a radar antenna in a site, calculation of SIR with this method is reasonable and practical. In these trends of deployments, the pattern of antenna deployment is predictable, making calculation of interference for or from a specific direction manageable. For a mobile wireless communication or a positioning system with a random location of the device, calculation of interference from multiple interferers becomes impossible demanding a method for empirical measurement and modeling of the interference (examples are available in Sect. 3). However, all spectrum management and regulations rulings have been based on this basic simple model for interference [9].

From a historical perspective, spectrum management and regulation have evolved from licensing the spectrum to enable a few commercial broadcasting services operate in populated urban areas and to separate their spectrums from those licensed for the military applications in communications, positioning, and navigation. With the growth of the wireless commercial and military applications the demand for spectrum increased and innovations in spectrum management and regulations began to emerge.

### Overview of Spectrum Regulation and Management

Established in 1934, the FCC manages the radio frequency spectrum in the United States for commercial and other non-federal uses, and since 1978 the National Telecommunications and Information Administration (NTIA) does the same for federal government use. In the commercial applications interference is unintentional and FCC regulates the spectrum by studying the interference for efficient utilization of spectrum and to managing coexistence among multiple services to satisfy the growth of demand by consumers. The exponential growth of commercial wireless communications industry relies on availability of more spectrum and innovations by FCC in regulations. In design of military applications, the main concern is on intentional interference that does not have anything to do with frequency management and regulations. However, NTIA coordinates with the FCC to make sure that FCC’s innovative regulations on spectrum management does not cause unintentional interference to military services operating in their licensed bands. In recent years interference from commercial wireless communications networks to the GPS has been a topic of long studies [13] because GPS enables the defensive and offensive capability of guided terrestrial and aerial systems that are the cornerstone of modern warfare.

Frequency regulation for commercial wireless networks, began in mid-1980s when FCC licensed 25 MHz of bandwidth for uplink and downlink to the first generation (1G) Advanced Mobile Phone System (AMPS) cellular networks for operation in the United States. The bands were granted with a traditional mask based on the simple SIR models for co- and cross-channel interference. Each band was shared among two regional service providers each with 12.5 MHz of bandwidth for down- and up-link communications. The bandwidth was managed for multi-user deployment with each service provider originally with omni-direction antennas and eventually with three sectored antennas. The exponential growth of the wireless industry in 1990s demanded increase of capacity from wireless cellular service providers. The industry began discovering new technologies to increase the capacity and at the same time service providers began negotiating with the FCC for additional spectrum. The research community responded with the TDMA followed by the CDMA technologies for the 2G and 3G cellular with a tenfold increase in capacity of cellular telephone and capability of supporting 2 Mbps data services. At that point, in mid-1990s, FCC began auctioning 20 MHz additional bands for approximately $20b for personal communication services (PCS). The high costs of the bands reflected the trade value of spectrum as a natural resource and how the government can auction that to promote more conscious usage of this scarce resource and to flourish the competitive growth of economy of the country in the cellular wireless networking. In these auctions the cellular wireless networking industry demonstrated its ability in generating capital for the growth of the economy. At the same time, auction of the new bands was allowing the cellular wireless providers to respond to the exponential growth of the subscribers to the cellular telephone services. However, emerging innovative wireless services such as the wireless local area networks needed much wider bandwidth, and they did not have economical resources to pay. This time FCC needed to come up with an innovative approach to interference regulation and management.

The importance of innovative regulation and management of spectrum was first experimented worldwide by FCC’s innovative release of Instrument Scientific and Medical (ISM) unlicensed bands in May of 1985 [2, 14]. The ISM bands are the first low-power unlicensed bands with restrictions on using spread spectrum technology to control and manage the interference at the devices. This first innovative step in spectrum management, nurtured the emergence of bandwidth hungry Wi-Fi technology and the IEEE802.11 standards for wireless local area networking to become the dominant technology for the wireless Internet access and a gateway to the smart world [3]. The unlicensed ISM bands became a public garden for experimental innovative wireless indoor technologies and in addition to the Wi-Fi for local area networking they nurtured other popular technologies such as Bluetooth and ZigBee for the personal area networking. The new emerging expensive bands such as PCS bands became privately owned smaller back yards with permissions for much higher transmission power to cover metropolitan areas for expanding a mature industry such as cellular with an exponential growth in revenue. The trend of low power transmission for unlicensed operation continued by release of UWB spectrum at 3.1–10.6 GHz in early 2000s and mm-Wave at 57–64 GHz in 2010s nurturing cyberspace commercial applications for short-range wireless communications and radars [7].

The next step in innovative spectrum regulations for high-power licensed band wireless cellular networks happened around 2010 by auctioning white spaces in the broadcast TV bands for other wireless applications on a priority basis. From a technical point of view this regulation introduces a new method of interference regulation management demanding monitoring the channel to examine the availability of a licensed band for other applications at different time and locations and it resulted in emergence of cognitive radio science and technology [15, 16] and a path towards the tumultuous liberation in spectrum management [17]. The trend continued into licensed priority access and spectrum monitoring for citizen band radio services (CBRS) [18], and C-band auction, originally assigned for satellite communications, Wi-Max, and Navy [19], followed by the adoption of L-bands in the vicinity of GPS bands, originally assigned for satellite communications. Currently, CBRS-bands are used for terrestrial wireless cellular networking application in a priority base, C-band auction is completed, and L-band is under evaluation for terrestrial use. In these priority spectrum management techniques, spectrum regulation mandates near real-time interference monitoring before transmission at the base station. In licensed bands ownership of the band is by a single service provider and interference monitoring is only applied by the service provider for deployment of the infrastructure in their own bands. In unlicensed bands interference monitoring is not mandated, but the medium access controls evolved for unlicensed bands (for example in IEEE 802.11 or 802.15.4) have adopted carries sense multiple access (CSMA) protocols, in which a devices senses (monitors) the channel before transmission as a libral method for interference management and co-existence.

The white space in the TV bands, CBRS, C-bands, and L-bands are under a few GHz and penetrate the walls making them a better suite for populated urban canyons and indoor operations, where most modern cyberspace applications occur. In parallel to discovery of these bands, the wireless communications networking industry is also discovering higher frequencies, where wider spectrum bandwidths are available. The mmWave bands are adopted for 5G and researchers are discovering THz and optical quantum communications for the future of wireless cellular networks. In these higher frequencies wireless networks should share the spectrum with astronomy radars and the understanding of the interference and methods for sharing the spectrum is under investigation [8]. Empirical interference monitoring and near real-time interference modeling is an essential component for efficient spectrum management and regulation and the future of the wireless cellular networks to enable sharing the spectrum at lower frequencies for larger cell and at higher frequencies for smaller cells.

## Different Views on Interference

Although FCC regulates level of acceptable co- and cross-channels interference with a similar interpretation of interference, the impact of interference on the performance of different sectors of the wireless communications and positioning industries are quite different. Performance of the communication systems is measured by maximum achievable data rate, or capacity, *C*, that is related to the bandwidth, *W*, the signal to noise ratio, SNR, and the selectivity of the antenna system expressed by the generalized Shannon-Hartley bound for MIMO systems [9]:

2a $$C \le W\sum\limits_{i = 1}^{N} {\log_{2} \left( {1 + \frac{SNIR}{N}\lambda_{i} } \right),}$$

where $\lambda_{i}$’s are the eigenvalues of the *N*×*N* cross correlation matrix of the *channel gains* between the elements of the transmitter and the receiver antennas. For $N = 1$ and the uncorrelated channels, $\lambda_{i} = 1$, this equation is reduced to the classical form of the Shannon-Hartley bound:

2b $$C \le W\log_{2} (1 + SNIR)\,$$

Performance of the positioning systems (GPS or Radars) are measured by ranging precision of the systems and the minimum variance of distance measurement error given by the Cramer-Rao lower bound (CRLB). The CRLB for a MIMO antenna system with an *N*×*N* transmitter antenna system is [6]:

3a $$CRLB = \frac{{48\sigma_{r}^{2} }}{{N(N^{2} - 1)\cos^{2} \alpha }},$$

where α is the angle of arrival of the signal, and $\sigma_{r}^{2}$ is the variance of the distance measurement error for omni-directional antennas, given by:

3b $$\sigma_{r}^{2} \ge \frac{{c^{2} }}{{8\pi^{2} \times SNIR \times W \times T_{M} \left( {f_{0}^{2} + W^{2} /12} \right)}},$$

in which *T*_*M*_ is the measurement time for calculation of the location.

As intuitively expected, both wireless communications and positioning system performances are improved with the increase in the bandwidth and a decrease in level of the interference. However, performance measurement criterion and details of derivations are quite different resulting in a diversified and complex relation toward spectrum management and regulations. We divide wireless industries into the wide and local area wireless communications and the positioning and radar systems to study their views of the spectrum management and regulations and analysis of the effects of interference. That complexity and diversity has created challenges in timely decision making for spectrum management by FCC and other agencies involved in regulation, causing delays in the economic growth of vital industries in the competitive wireless communications industry. We proceed with an overview by examples to give a broad understanding of the vital issues in spectrum regulation and management in different sectors of the industry.

### Interference in Wireless Cellular Networks

Among all wireless communications industries, cellular wireless grew for wide area operation in licensed bands with mainly outdoor antenna deployments. For this industry “spectrum is not only the lifeblood but also influences 5G supply chain integrity, economic growth, and national security [13]”. Cellular wireless services support comprehensive coverage, high mobility for operation in the vehicles, controlled delays for the time sensitive applications, and they face an exponential growth of demand for more bandwidth. The cellular wireless service providers own a portfolio of licensed bands and deploy their base stations based on frequency reuse and a centralized co-channel interference control and management. The deployment of cellular infrastructure is based on a cell hierarchy with different cell sizes. Smaller cells operate with lower power and support higher capacity per unit of bandwidth with lower mobility. Larger cells operate at higher power and support lower capacity per unit of bandwidth with higher mobility. The interference generated by small cells is more uniformly distributed over the area of coverage. The traditional cell hierarchy includes macro-cells with a coverage of a few Km with a typical transmitted power of 40 W, micro-cells with 500 m coverage and 5W transmitted power, pico-cells with 200 m coverage and 2 W power, and femto-cells with 30 m coverage and 100 mW power [20]. All cellular base station antennas are deployed outdoors, except for some femto-cell antennas that are deployed indoors. In indoors, femto-cells compete with Wi-Fi, which carries 70% of the total IP traffic without a need for subscription to a cellular service provider [7]. These very small cells transmit at low power of approximately 100 mW and they are barriered by an additional 10–20 loss to penetrate to outdoors containing their interference inside the building. As a result, the impacts of interference from other cells with outdoor antenna deployments are more and as the size of cell and consequently transmitted power level increases, they raise more concerns on cross-channel interference with other licensed spectrums in their neighboring bands. With the recent trends in releasing satellite bands for terrestrial applications that we discussed in Sect. 2.2, there is a need for understanding the impact of interference from the cellular wireless networks operating in the L bands neighboring to the GPS bands [13, 21].

Wireless cellular service providers are not concerned with out of band interference from others operating in their neighboring bands, they are concerned with co-channel interference control for frequency reuse and efficient spectrum management for deployment of the cellular infrastructure. Cellular networks are interference limited networks, and their capacity increases with reduction of the interference. However, this interference is co- and cross channel interference in the bands that they own. Traditionally, service providers calculate SIR for the frequency reuse factor (*N*_*f*_) from [9]:

4 $$SIR = \frac{1}{{J_{s} }}\left( {3\sqrt {N_{f} } } \right)^{\alpha } ,$$

where α is the distance power-gradient of the environment,^1^*J*_*s*_ is the number of interfering neighboring cells that is a function of the spatial selectivity of the antennas. For omni-directional antennas $J_{s} = 6$ and for the three sector antennas, $J_{s} = 2$. In the 4G and 5G cellular and beyond macro-cells are deployed by frequency reuse factor of three and smaller cells with frequency re-use factor of one in an overlay-underlay setting where the interference among smaller cells is controlled by physical separations beyond their coverage areas [9]. The huge MIMO antenna systems with very narrow beamforming capabilities theoretically can eliminate the interference allowing availability of the entire spectrum for each individual end user. As a result, in 5G they migrated to massive MIMO and for 6G they study super-massive MIMO antenna systems for their capabilities in producing narrower beams and to support higher number of streams [9].

Today, cellular service providers own a portfolio of bands at different frequencies with different power and priority restrictions. To expand this portfolio the cellular wireless industry has also resorted to higher frequencies, the 5G industry discovered the mmWave and THz technologies are emerging for 6G and beyond. These bands share the spectrum with radars for the military and commercial applications in a variety of bands for environmental monitoring and astronomy and that has initiated research studies in the interference caused by cellular industry into these industries [8]. Since in these frequencies RF signal does not penetrate the walls, the wireless industry also have resorted to spectrum sharing with other license spectrum holders at lower frequencies for applications demanding through the wall penetrations. Spectrum sharing demanded interference monitoring and cognitive radios, and it began with the white bands for broadcasting services, followed by C-band for satellite communications, and L bands with potential interference with neighboring GPS bands. This situation initiated a momentum for research in the interference monitoring science and engineering to enable a new foundation for decision making and intelligent spectrum management. What is important for the cellular wireless industry is more efficient use of the spectrum in time, frequency, and space in metropolitan areas with high density of population. To increase the efficiency, we need to monitor the interference in the available spectrum to enable spectrum sharing. Analysis and understanding of the nature of interference and near real-time interference assessment for intelligent spectrum management is an important area for research and developments for the US cellular wireless industry to maintain its competitiveness and security.

### Interference in Wireless Local and Personal Area Networks

The ISM bands released in May 1985 were the first unlicensed bands with low-power transmission (less than oneWatt) that also enforced spread spectrum transmission with a minimum processing gain of ten to further control the interference to other devices. The IEEE 802.11 Wi-Fi for local networks followed by IEEE 802.15 Bluetooth for personal area networking were the leading popular commercial standards emerging in these bands with the spread spectrum technology in late 1990s. The maximum transmission power of Wi-Fi and eventually Bluetooth are 100 mW and both are mainly deployed indoors where exterior walls provide a 10–20 dB shield that further contain the interference to indoors. The background of spread spectrum radio designers that were engaged in that emerging industry was in design of spread spectrum systems for military applications to counteract the effects of intentional interference from a variety of jammers. Unlicensed bands were released to nurture commercial application developments where interference is unintentional. However, designers of early spread spectrum devices in ISM bands were coming to the field with past military design applications and they were paranoid from the effects of interference rooted in the history of design of spread spectrum communications systems for military applications. A review of some of these experiences as examples may help future spectrum regulations when military and commerce come with different interpretation of interference in disputable bands such as L bands.

In the first few years of the IEEE 802.11 standardization process,^2^ they were deciding on 2.4 GHz bands as a better option over 900 MHz, because 2.4 GHz was available worldwide and it had wider bandwidth (84 MHz at 2.4 GHz vs 26 MHz at 900 MHz) allowing more alternative channels for communications. One of the issues working against adoption of 2.4 GHz that was discussed in length in the early IEEE 802.11 committee meetings was interference from microwave ovens. Researchers at Hughes Network Systems, San Diego, a military communication company keen on discovering commercial applications for military communication technologies, had a thorough study of characteristics of interference from microwave ovens to present to the IEEE 802.11 community [22, 23]. The IEEE 802.11 finally decided on 2.4 GHz, neglecting the significance of interference from the microwave ovens reported to the committee. Today, many of us use Wi-Fi in our kitchens and next to our microwave ovens and some microwave ovens are remotely controlled by Wi-Fi. This example reflects that unlike intentional interference in military communications that we design our systems to manage them, in commercial unintentional interference operation in the liberated unlicensed bands interference exists but we live with it without any special effort.

The second time that interference in *unlicensed bands* attracted attention was at the emergence of the IEEE 802.15.1 Bluetooth technology in late 1990s, right after finalization of the IEEE 802.11 and emergence of the IEEE 802.11b as the first successful Wi-Fi technology capturing the sizable small business and home office (SOHO) market for wireless Internet access. At that time many progressive large corporates, such as Fidelity Headquarter in Boston, had invested in deployment of IEEE 802.11b infrastructure to enable their task force to carry their laptops within the corporate buildings as well as to their home. The executives behind these decision makings and financial investments became panicked of the effects of interference from Bluetooth to jeopardize the company’s investments. Again, at that time, most technical people behind the decisions had military communication background and were paranoid of interference and its impact on security of the network. This time the scenario for paranoia was strong enough and more realistic that it initiated the IEEE 802.15.2 to address the interference and discover methods for co-existence between Wi-Fi and Bluetooth in 2.4 GHz ISM bands. Although the transmission power of the IEEE 802.11b was 20 dBm (100 W) and the Bluetooth in US operated with 0 dBm (1 mW) to control that interference, understanding of this new interference scenario in unlicensed bands and discovery of a standard guideline to help co-existence in unlicensed bands became the charter of the IEEE 802.15.2.

The interference scenario and method for the analysis of interference for the IEEE 802.15.2 was fundamentally different from the scenario for interference analysis in cellular networks. In cellular networks a service provider deploys the infrastructure in a licensed band following a specific frequency reuse pattern that is related to the transmission technology, the service provider manages the interference among devices centrally, and all devices use the same transmission technology. In the IEEE 802.15.2 interference scenario, deployment is random and often by different entities without much of coordination for interference management, and transmission technologies for different devices are different. The IEEE 802.15.2 began its study by defining an application scenario for this complex and diversified interference analysis problem involved in SIR as well as details of different transmission techniques recommended by the IEEE 802.11 Wi-Fi and the IEEE 802.15.1 Bluetooth.

**Fig. 2:**
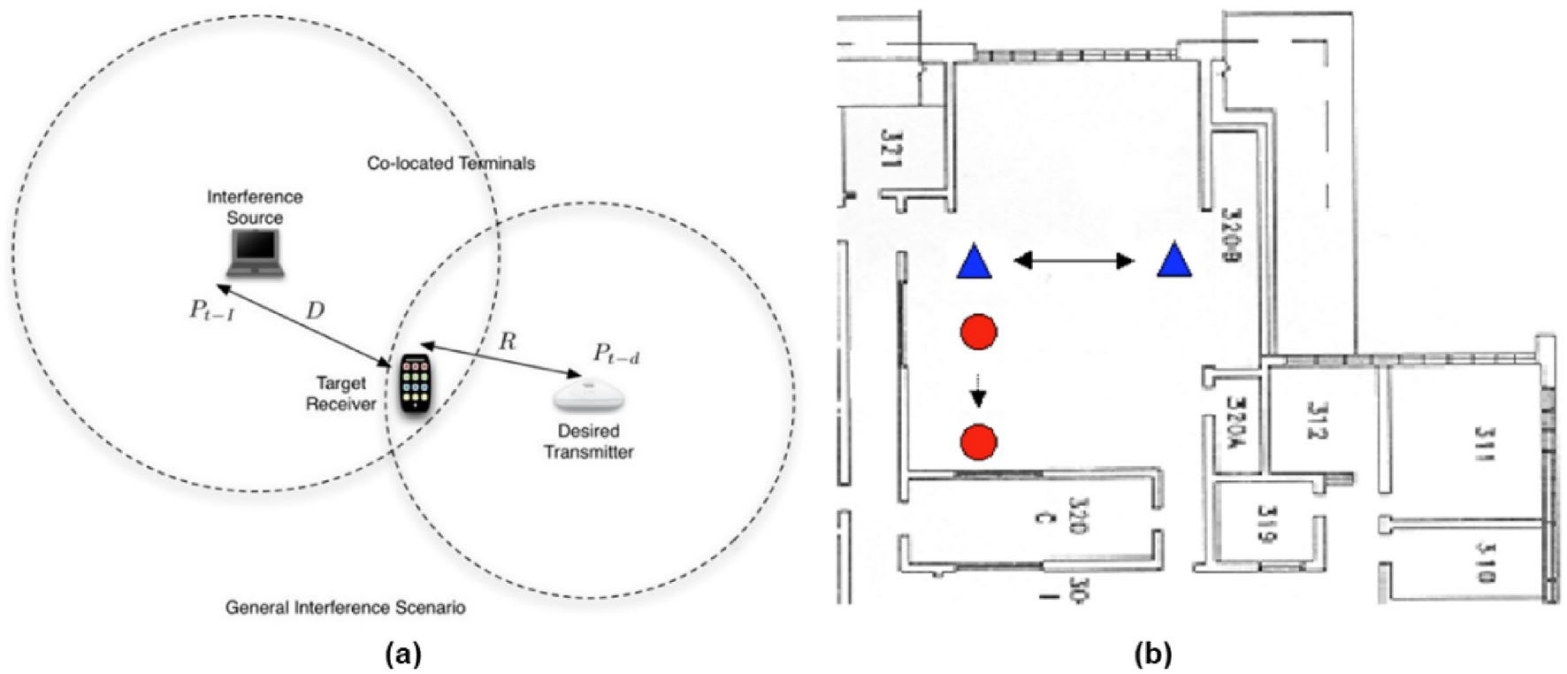
Interference scenario between Wi-Fi and Bluetooth in IEEE 802.15.2, **a** the basic concept, **b** a practical setting at CWINS laboratory, third floor of Atwater Kent Laboratories, WPI, Worcester, MA [24]

**Fig. 3:**
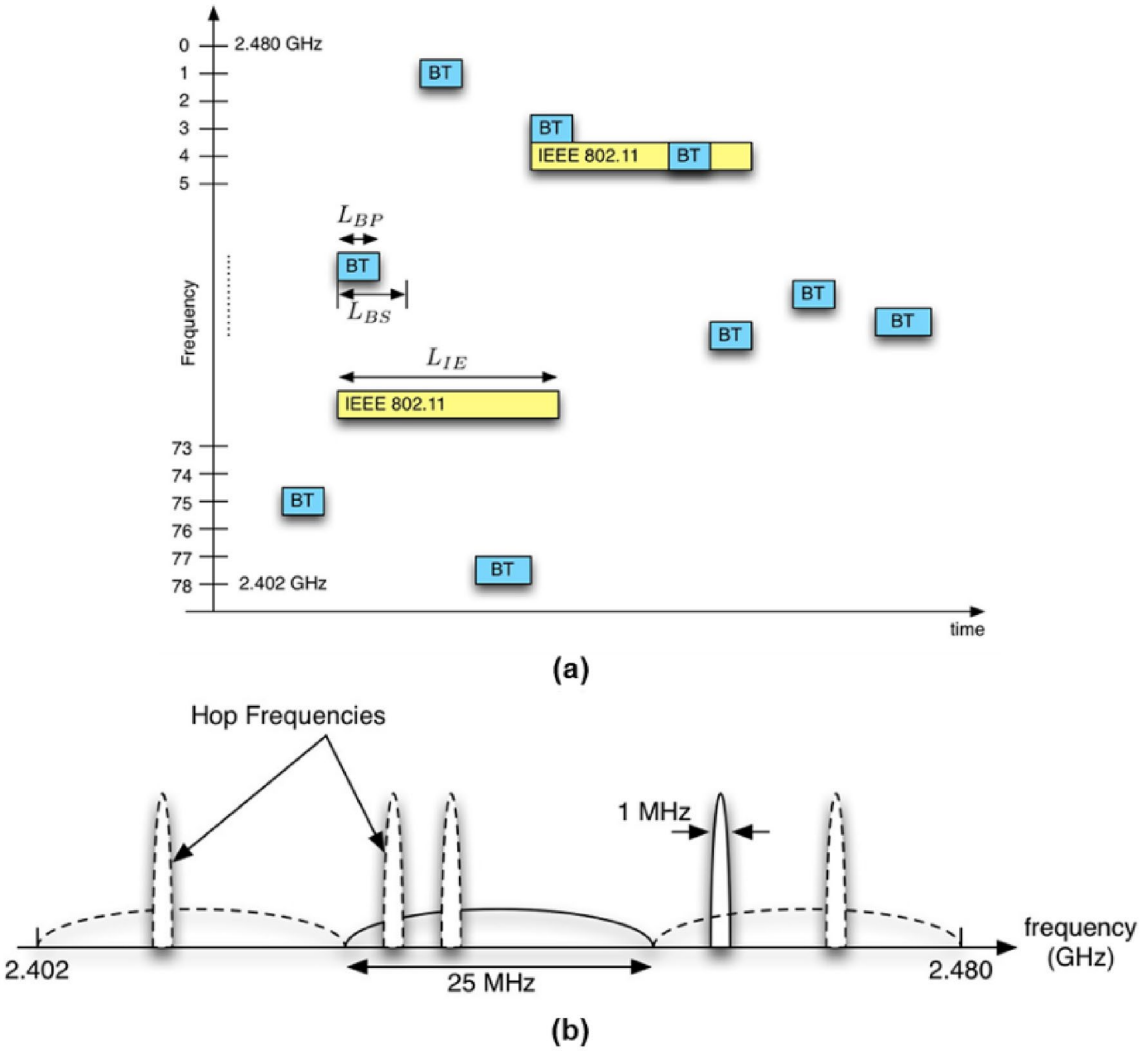
**a** Interference in time-domain between FHSS IEEE 802.11 and Bluetooth (BT), **b** interference between DSSS IEEE 802.11 and BT in frequency domain [25]

Figure 2a shows the basic concept of the interference scenario defined by the IEEE 802.15.2, for this scenario we can calculate the SIR from Eq. (1b) using transmitted power and distances of desired device, *P*_*D*_, *R,* and interfering device, *P*_*I*_, *D*. Figure 2b, shows a typical implementation of this scenario in the CWINS laboratory at the third floor of the Atwater Kent Laboratories, Worcester Polytechnic Institute, Worcester, Massachusetts [24, 25]. With this scenario we can determine how close two devices should get to interfere with one another. However, to measure the impact on the user experience, the IEEE 802.15.2 began analysis of the effects of interference of each device in increasing the packet error rate of the others. This analysis goes beyond the coverage calculation of each device and gets engaged with the details of signals transmitted from the devices in the time and in the frequency. Figure 3a shows a typical time-domain interference scenario between long packets of the FHSS IEEE 802.11 and Bluetooth’s shorter packets and Figure 3b shows the interference scenario between IEEE 802.11b (or DSSS 802.11) with 26 MHz bandwidth and 1 MHz Bluetooth bands with random hops. These examples reveal the complexity of the interference analysis in the time- and in the frequency-domain. Considering differences in the medium access methods adopted by different technologies add to this complexity demanding more in depth analysis and empirical measurement studies to understand the real impact of interference in unlicensed bands.

One of the early studies in this area to analyze the interference among IEEE 802.11b and the Bluetooth (Fig. 3b) is reported in [24]. This study uses the IEEE 802.15.2 scenario in Fig. 2b to analyze the effects of mutual interference among the two wireless communications technologies and demonstrates that calculation of interference with antenna power from Eq. (2b) is far more pessimistic than that of empirical measurement of packet error rate (PER) like those in Fig. 3 [24]. From these observations, we can conclude that analysis of interference in liberated unlicensed bands is a complicated problem and the correct approach for a realistic solution involves empirical measurement of PER while SIR provides a bound on how close the two devices should be to interfere with each other. The difference of calculation of interference base on SIR and the PER becomes more complex as we include the effects of MIMO antenna systems with beam forming capabilities. In these cases, the angular orientation of antennas also plays a role in the interference. An example case study for the analysis of interference among wireless technologies using MIMO antenna systems is available in [26]. This study analyzes the mutual interference among IEEE 802.11ad wireless communication devices and the short range mmWave radars both benefitting from MIMO antenna systems and operating in unlicensed mmWave 60 GHz bands. As we explained earlier, wireless communication performance is reflected by Shannon-Bounds (Eq. 2), while performance of positioning systems is measured by CRLB (Eq. 3) posing another challenge for analysis of the effects of interference on the user applications of wireless devices. The study presented in [26] reveals that while the interference from mmWave radars to IEEE 802.11 communications devices affects the PER of the communication device, the interference from the IEEE 802.11 communications devices only reduces the range of coverage of the mmWave radar without any significant effect on its precision.

The IEEE 802.15.2 arrived at recommendations for co-existence in 2003 and later these recommendations were withdrawn [27]. However, studies of this committee revealed the fact that the simple SIR analysis cannot lead to a realistic understanding of the effects of interference in non-homogeneous wireless communications devices operating in liberated unlicensed bands. They addressed the complexity of this problem and they proposed a methodology for empirical modeling of the interference, and among all they also recommended listen before talking (LBT) as a protocol for operation in these environments. The LBT was implemented in CSMA based protocols of the IEEE 802.11 before and in the IEEE 802.15.4 (the ZigBee) later. These studies also pointed to the need for a new theoretical foundation for interference analysis to enable intelligent spectrum management.

Another notable mis-understanding on the nature of interference affecting FCC regulation on unlicensed bands occurred in early 2000s, when UWB technology emerged for low power transmission and precise ranging. In 2002 FCC released the UWB unlicensed bands at 3.1–10.6 GHz to enable this technology studied by the IEEE 802.15.3a [9]. The lead UWB company, the Time Domain Corporation, Huntsville, Alabama, had implemented their technology with baseband pulses with super low power density across all other licensed and unlicensed applications in the first 5 GHz of spectrum. The GPS community came with a strong statement against the role making arguing that GPS operates in very low power and UWB can potentially damage its performance. As a result, FCC changed its spectrum profile and included a notch in the GPS and its neighboring bands. Today, UWB products emerged in these bands are mainly used for precise indoor positioning and the GPS does not work indoors with or without interference from UWB devices. May be FCC could allow the UWB operation in full band only for indoor applications.

### Interference in Positioning Systems

In Sects. 3.1 and 3.2 we provided a review of the way that interference has been studied for wireless communications in licensed and unlicensed bands to explain the nature and complexity of understanding the real impact of interference in different wireless communications systems designed for military and commercial applications. We also provided a few examples of the impact of misunderstanding of the difference between intentional interference in military applications and unintentional interference in commercial applications causing delays in commercial developments. In this section and the next we review the effects of interference in the two classes of localization technologies: the active positioning systems, and passive radars.

Performance criteria for localization systems is the precision of positioning reflected by the variance of the distance measurement error calculated from the CRLB given by Eq. 2. The CRLB is a function of bandwidth, SNR, and the measurement time that means if we change the SNR two times (3 dB) precision or standard deviation of error will increase 1.43 times. Equation 2 also reflects that we can compensate for that 3 dB loss of signal power by doubling the measurement time. That means if we have a location fix in one second and we increase it to two seconds, we compensate for a 3 dB loss of power. Another alternative to compensate for the precision is to double the bandwidth and we already know how expensive it is. Waiting for the location fix is very important in automated tracking systems such as autonomous driving vehicles and automated weaponry systems. In most other military and commercial positioning and navigation applications we can often compromise on the less expensive waiting time.

The first popular positioning system for military, public safety, and commerce, the GPS, covers approximately 99% of the vast outdoor areas on the globe [4] but not indoors, where approximately 80% of the IP traffic is generated to enable smart world applications [7]. In open outdoor areas, the GPS can achieve precision of approximately 1m for military applications, but in multipath urban areas, where all the smart world popular applications take place, GPS has a precision of 10–15 m [5]. The significant performance degradation of GPS in these environments is due to the extensive multipath conditions. The other two popular positioning systems, Wi-Fi positioning systems (WPS) and cell-tower positioning systems (CPS) [5, 6], emerged for coverage in indoors and outdoors. The WPS is the most popular in smart world applications and it has a precision of 1–15 m depending on availability of indoor fingerprints in the databases of the system [5]. The WPS works in major populated indoor and urban areas, but not in highways and vast open areas. The CPS extends the coverage of the WPS to highways and open areas but the precision of its existing technologies are substantially lower [6]. With the popularity of MIMO antennas and with narrow beam forms in 5G and beyond, precision of the CPS systems are expected to improve significantly.

In the world of commerce, the three positioning technologies, GPS, WPS, and CPS, complements each other based on availability of these technologies in the platform of operation. Today, several billions of smartphones worldwide carry all three positioning technologies, computing equipment (tablets, laptops, PCs), as well as smart devices (TVs, security systems, and IoT devices) do not have cellular or GPS chipsets and by default they benefit only from WPS. The largest commercial market for the GPS grew after its integration in the second generation iPhone. The first generation of iPhone was only relying on WPS and CPS [28]. Each smartphone enables millions of software applications and most of these applications benefit from a sort of positioning for a variety of applications covering classic turn-by-turn navigation, as well as searching for services from Google or Yelp [29]. Majority of these applications benefit from Wi-Fi positioning data bases and for that reason the databases, such as the original Skyhook database, receive over a billion hits per day [3]. However, still turn-by-turn navigation remains as the most popular positioning application in smartphones and outdoor turn-by-turn navigation relies on GPS as the first option.

The GPS operates in its own licensed L bands and the US Government funded the expensive GPS satellite infrastructure because of its importance for military applications. The WPS and CPS are opportunistic positioning systems benefitting from the existing infrastructure of Wi-Fi access points operating in unlicensed bands and cell-towers infrastructure for wireless communications operating in privately owned licensed bands. The GPS is the heart of navigation of aerial and terrestrial vehicles for commercial and military applications and it is used for many commercial applications in wide open areas. Military and public safety applications give a special weight to GPS technology and maintenance of its precision for guided missiles and drones. Mitigation of intentional interference to the GPS has been an area of research for military applications for many years. Recently, effects of unintentional interference from neighboring wireless communications systems operating in L bands has been under investigations. The WPS and the CPS technologies emerged for commercial applications and they adjust live with the interference.

The GPS is tied with the precision tracking for national security and military applications to direct missiles and drones to targets and it has found its way in a variety of commercial applications in avionic and space, robotics and machine control, agriculture, scientific, timing, and survey and mapping. Analysis of interference in GPS has turned to a complicated problem with technical complexities, economic considerations, and political concerns [13, 21]. To help this situation, it is beneficial to monitor the interference in GPS bands in indoor and urban areas as a scientific endeavor for potential research on impact of interference and comparative evaluation of the effects of multipath on intentional and unintentional interference in GPS for hostile applications in urban areas. The frequency administration agencies can benefit from the better understanding of the effects of interference to come up with new methods for intelligent interference regulation and management to avoid un-necessary role making. As we explained in Sect. 3.2, the IEEE 802.11 and the IEEE 802.15 had experiences in this domain that should not be neglected. Having a theoretical foundation for analysis of interference can resolve many of these discrepancies and uncalled for paranoic interpretations of interference. The GPS manufacturers can also tighten the sharpness of their front end receivers to better controls the cross-channel interference from neighboring bands.

### Interference in Radars

**Fig. 4:**
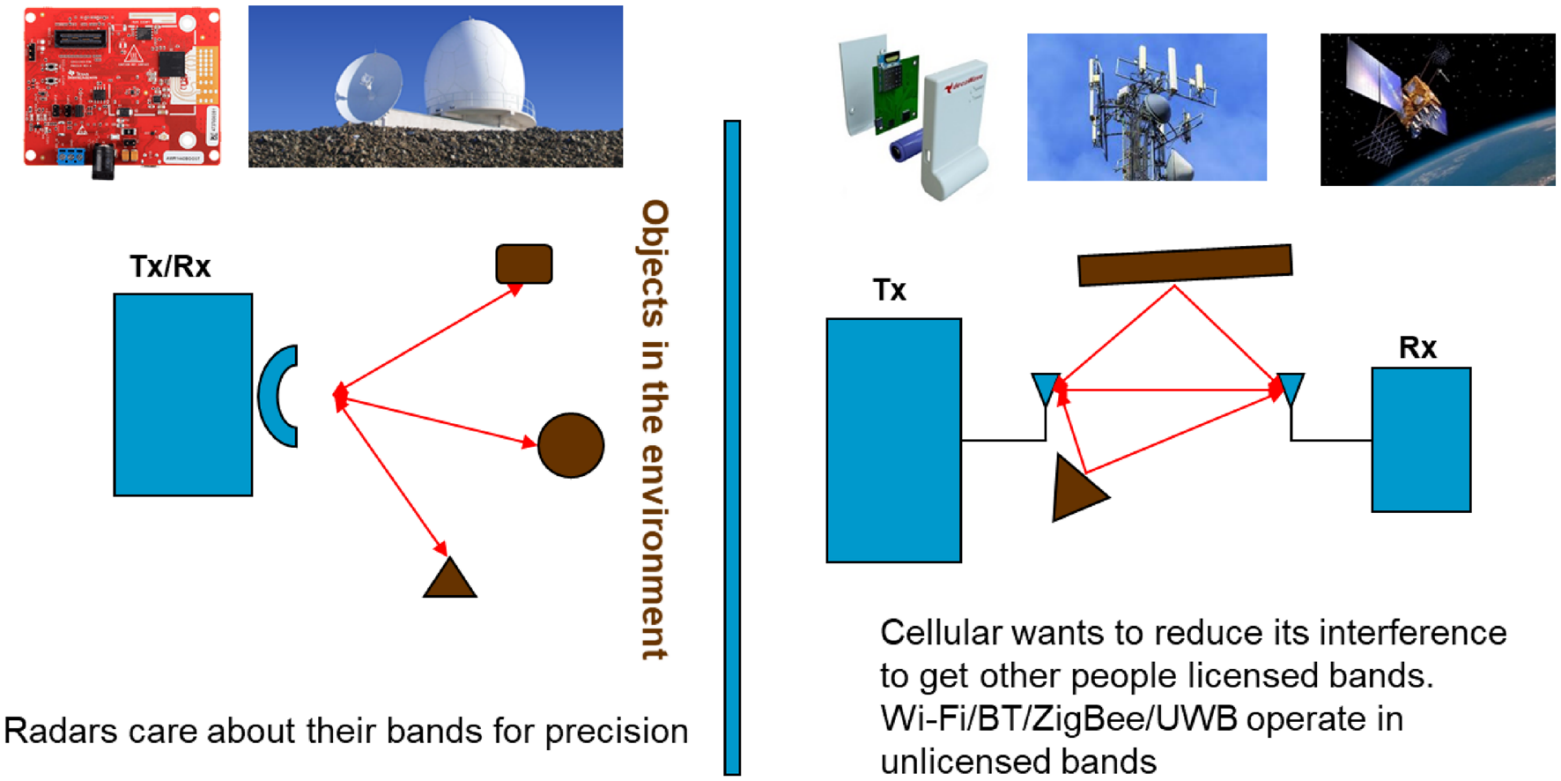
Comparison of radar with wireless communications and positioning systems

Radar was invented for military applications during the WW-2 and currently they operate in a variety of traditional licensed spectrums applications for locating distance objects in astronomy, navigation, imaging, and environmental monitoring [10]. Recently, short range mm-Wave and UWB radars operating in unlicensed bands becoming popular for emerging cyberspace applications in human-computer interaction, gesture and motion detection, and authentication and security [3]. Like any other wireless access and localization systems, radar has a transmitter and a receiver, but transmitter and receiver are in the same location. Therefore, radars are also subject to the cross- and co-channel interference. Like positioning systems, the objective of a radar is to position location of a targets. As shown in Fig. 4, the difference between wireless positioning systems and the radars is that in the radar the target does not participate in positioning making radar a passive positioning system.

Like positioning systems, performance of a radar is measured with the CRLB and precision of positioning (Eq. 2) and it is affected by interference, bandwidth, frequency of operation, and the measurement time. Radars are more sensitive to interference than positioning systems because the received signal is reflected from the target making it a much weaker signal. Most popular radars operate with MIMO antenna systems with beamforming to position the targets with the range and the angle of arrival of the signal reflecting from the objects. As we discussed before, directional antennas create an embedded mechanism to reduce the interference from other devices as well as interference they cause in other devices. Radars sweep the space in different angles periodically with a gap time between sweeps to save in transmitted power. This feature makes radars look like an impulse interference source to other wireless devices.

Long range radars operating in licensed bands are popular in passive navigation and tracking of aeronautical and terrestrial object in military and commercial systems. A unique application of radars is in astronomy, in which the objects are far away, the received signals are very weak, and interference sources are much closer than the targets. At the same time, many of these radar systems do not fully utilize their licensed bands in time and in the terrestrial locations. In the commercial world the bandwidth hungry wireless communication networks are eager to share these bands with them but that demands a realistic understanding of interference. A near real time interference forecasting system helps resolving this situation and opens a horizon for discovery of more innovative and realistic algorithms for intelligent spectrum management for these applications.

A theoretical analysis of interference between wireless communications devices and radars have been a challenging problem. Thanks to the growth of wireless networking industry, recently low cost miniature UWB and mmWave radars in unlicensed bands have emerged in the market. We have miniature short range radars at 3–10 and 60–70 GHz that operate in unlicensed bands that are available for scientific study of the effects of interference between wireless communications devices and the radars to lay a theoretical foundation for understanding the nature of interference among communication and positioning systems. In Sect. 3.2 we provided an example study in this area for the analysis interference between IEEE 802.11ad and the mmWave radars operating at 60 GHz [26]. Using these devices, we can also study the UWB radars and IEEE 802.11ac/ax operating in 3–10 GHz spectrums and many other scenarios to lay a theoretical foundation for RF cloud interference analysis.

## Theoretical Foundations for RF Cloud Interference Modeling

Better understanding of characteristics of the RF cloud interference is necessary to enable the emerging new paradigms for intelligent and liberated spectrum management and regulations [8, 17]. This challenge demands a theoretical foundation for a near real-time RF cloud interference monitoring and forecasting. The interference analysis and design of counter-interference measures for RF military communications, positioning, and navigation applications has been a subject of decades of research. The research objective for these applications were the design of robust communications, positioning, and navigation systems to counter the effects of un-controllable intentional short-time interference [10]. The nature of interference in military applications is intentional, interference sources are often limited in numbers, and the period of interference is limited to the duration of military conflicts or disaster recoveries. In the commercial world interference is unintentional, we have unlimited interfering devices, and the duration of interference has no limits in time.

As we explained in Sect. 2.2, the exponential growth of commercial RF devices demanding more bands lead to the request from wireless communications industry for sharing some of the exiting terrestrial and satellite bands previously assigned to other military and commercial applications based on new priority schemes. Implementation of priority schemes demanded technologies for near real-time interference measurement and modeling in these bands. The objective of interference analysis in commercial world is to optimize utilization of spectrum for intelligent interference management to increase the capacity of wireless communication networks. Science and technology for the analysis of RF cloud interference for intelligent spectrum management is at its infancy and it demands a theoretical foundation to begin a meaningful growth. Here we begin with an analytical study beneficial for analysis and simulation of the interference in an IoT environment, then we propose a method for implementation of an empirical intelligent interference forecasting system (IIFS) with a centralized database for near-real-time interference monitoring and forecasting for the future of all wireless devices.

### Modeling the Short Term Temporal Behavior of the Interference

**Fig. 5:**
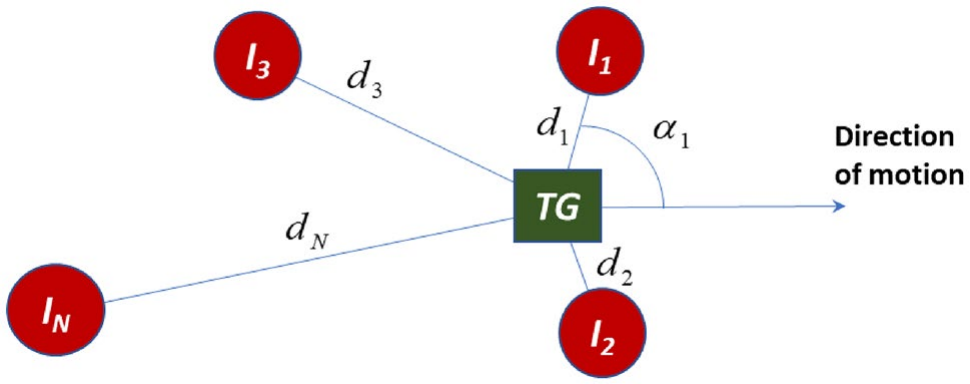
Interference scenario for N-interferes with uniform distribution of their distances and their angle with respect to the direction of motion of the target device

We can analyze the short term variations of the interference in an IoT environment where many stationary and moving wireless devices interfere with each other based on circular scattering principles. This modeling enables us to have a realistic performance evaluation of the RF cloud interference with any wireless device. Figure 5 illustrates an interference scenario for *N*-interferes, uniformly distribution around a target receiver (TG), each with a distance $d_{i}$ and a physical angle $\alpha_{i}$ with direction of movement of the device. The RF propagation analysis for this scenario resemble the RF multipath propagation with circular scattering for wireless communications applications [12, 30]. In circular scattering for wireless communications the transmitted signal arrives at the receiver from multiple random paths reflected from surrounding objects circling the receiver. In interference modeling scenario shown in Fig. 5 multiple interfering signals arrive randomly from multiple IoT devices surrounding the target receiver (TG). Defining the amplitudes and phase of the received carrier signal from each of the *N*-interferes by $A_{i} \,\,{\text{and}}\,\,\phi_{i}$, respectively, the amplitude, phase, and power,$A_{I} \,\,,\,\,\phi_{I} \,,\,P_{I}$ of the interference signal at the target device are:

$$\left\{ \begin{gathered} A_{I} e^{{j\phi_{I} }} = \sum\limits_{i = 1}^{N} {A_{i} e^{{j\phi_{i} }} } \hfill \\ P_{I} = \left| {A_{I} } \right|^{2} \hfill \\ \end{gathered} \right..$$

According to the central limit theorem the distribution function of the in-phase and quadrature phase components of the interference are Gaussian, therefore the distribution functions of amplitude, phase, and power of the interference follow the Rayleigh, uniform, and exponential distributions, respectively [12]:

5a $$\left\{ \begin{gathered} f_{\phi } (\varphi ) = \frac{1}{2\pi };\,\,\, - \pi < \varphi \le \pi \hfill \\ f_{A} (a) = \frac{a}{\Gamma }e^{{ - a^{2} /2\Gamma }} \hfill \\ f_{P} (p) = \frac{1}{{\overline{{P_{I} }} }}e^{{ - p/\overline{{P_{I} }} }} \hfill \\ \end{gathered} \right..$$

In this equation $\Gamma$ is the mean-square amplitude of the arriving signal, and $\overline{{P_{I} }}$ is the average received interference power. Knowing the distribution function of power of the interference, we can calculate the average capacity of a communication device with omni directional (OD) and MIMO antennas by integrating Eq. (2a, b) over the distribution of power given in Eq. (5a):

5b $$\left\{ \begin{gathered} \overline{C}_{{{\text{OD}}}} \le \frac{W}{{\overline{{P_{I} }} }}\int\limits_{0}^{\infty } {\log_{2} \left( {1 + \frac{{P_{d} }}{{N_{0} W + p}}} \right)} \,e^{{ - p/\overline{{P_{I} }} }} dp\,\,\, \, \hfill \\ \overline{C}_{{{\text{MIMO}}}} \le \frac{W}{{\overline{{P_{I} }} }}\int\limits_{0}^{\infty } {\sum\limits_{i = 1}^{M} {\log_{2} (1 + \frac{{P_{d} }}{{N\left( {N_{0} W + p} \right)}}\lambda_{i} )} } \,e^{{ - p/\overline{{P_{I} }} }} dp\,\, \hfill \\ \end{gathered} \right..$$

Similarly, precision of a positioning system with omni-directional antennas and MIMO antennas is obtained by integrating Eq. (3a, b) over the distribution of the interference power:

5c $$\left\{ \begin{gathered} CRLB_{OD} = \overline{{\sigma_{r}^{2} }} \ge \frac{{c^{2} }}{{8\pi^{2} \times W \times T_{M} \left( {f_{0}^{2} + W^{2} /12} \right) \times \overline{{P_{I} }} }}\int\limits_{0}^{\infty } {\frac{{N_{0} W + p}}{{P_{d} }}} \,e^{{ - p/\overline{{P_{I} }} }} dp\,\, \hfill \\ CRLB_{MIMO} = \overline{{\sigma_{r}^{2} }} \ge \frac{{48 \times \overline{{\sigma_{r - OD}^{2} }} }}{{N(N^{2} - 1)\cos^{2} \alpha }}\,\,\,\,\, \hfill \\ \end{gathered} \right..$$

As a device moves along a path with velocity *v*_*m*_ the Doppler shift from each interfering source depends on the spatial angle of the direction of movement with the direction of the source, α

$$f_{d} = \frac{{v_{m} }}{\lambda }\cos \alpha = f_{m} \cos \alpha \,\, \Rightarrow \,\,\alpha = \cos^{ - 1} \left( {\frac{{f_{d} }}{{f_{m} }}} \right).$$

For uniform distribution of interference angle, α

$$f_{\rm A} (\alpha ) = \frac{1}{2\pi }\,\,;\,\,\,\,\,\, - \pi < \alpha \le \pi,$$

the Doppler spectrum of the interference is [12]:

5d $$D\left( f \right) = f_{\rm A} (\alpha ) \times \left| {\frac{d\alpha }{{df}}} \right| = \frac{1}{{2\pi f_{m} }}\left[ {1 - (f/f_{m} )^{2} } \right]^{ - 1/2} ;\,\,\,\,\left| f \right| < f_{m} \,.$$

Equation (5c, d) allows one to simulate the interference for a mobile user for performance analysis by running a complex Gaussian noise through a filter reflecting Doppler spectrum characteristics [12]. Then we can design software and hardware interference simulators to examine the effects of IoT interference on a communication link, a GPS device, or a radar.

### Empirical Modeling of the Near Real-Time RF Cloud Interference

In the previous section we provided an analytical model for the temporal behavior of the RF cloud interference and methods to simulate their variations for mobile terminals when we know the average interference power in a location $\overline{{P_{I} }}$. Because of random distribution of location and power levels of interfering wireless devices, randomness in shadowing from objects surrounding them, diversity, and spatial selectivity of antennas for different devices, and randomness in motion of the devices, we need a near-real-time empirical interference monitoring system to monitor and forecast the $\overline{{P_{I} }}$ in different locations. With a near real-time estimate of $\overline{{P_{I} }}$ in all locations, we can optimize utilization of spectrum in that location. A near real-time interference prediction system needs a centralized interference monitoring database to interact with wireless devices to help them optimize the spectrum sharing for wireless communications and to minimize their interference with positioning and navigation systems.

**Fig. 6:**
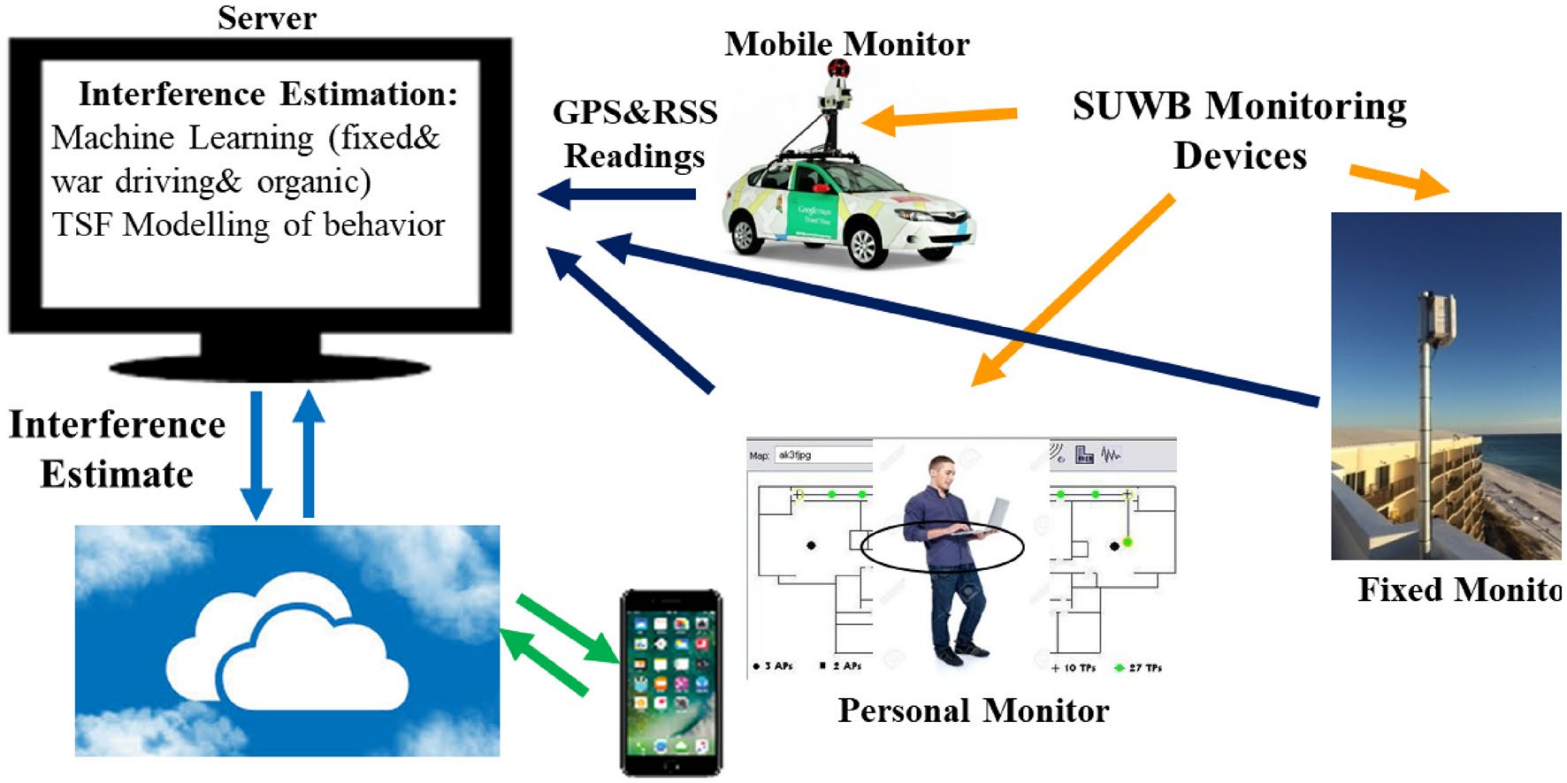
General architecture of the IIFS showing the elements of the system for creation of the database

The existing near real-time interference monitoring systems for spectrum management monitor the interference for fixed base stations to enable spectrum sharing with priorities [31]. Figure 6 shows a proposed architecture for a cyber physical system for empirical interference monitoring for near real-time intelligent interference forecasting, the IIFS [32]. A multi-band programmable scanning software defined radio tuned to all active frequency bands in a region measures the received signal strength from interfering devices, $\overline{{P_{I} }}$. Multiple driving vehicles equipped with multi-band scanning devices measure the time-frequecy characteristics of the interference in a location, and stamp the measurement with a GPS/WPS/CPS positioning engine estimate of that location. The location stamped measurement are then transferred to a central computing server. The target surveying region is traversed in a programmatic route to avoid arterial biases. The programmatic route includes all drivable streets in the target geographical area following the same driving method for fingerprinting in WPS positioning systems [5, 33, 34]. At the server, location, time, and frequencies of the measured $\overline{{P_{I} }}$ from different angles of arrival are used to train a machine leaning algorithm such as GAN to create a fingerprint map for the interference in the area [35, 36] and to predict the future interference expectation in time-frequency and location. A user-device with a RF radio platform with cellular, Wi-Fi, Bluetooth, or other emerging egresses, receives the $\overline{{P_{I} }}$ of its location from the server to find the optimum egress RF channel for its application, for example to stream a video, establish a telephone conversation, transfer a file, or browse the web.

In time the miniature version of multi-band receivers will integrate into user devices to monitor the interference and make real time spectrum management at the device. These devices report their measurements to the central server to update the database and increase the accuracy of the IIFS database. The database originally created by war driving will update itself with this organic data collected from users as the application grow in time. The need for war driving will reduce in time as the organic data increases by popularity of the database. Prior to expanding the database with war driving with vehicles, the public data on interference measurement programs sponsored by NSF and other government agencies will be fed to the database. The reference database of predicted interference levels of RF devices in each location in a target area is kept in the Edge Cloud to be accessible to all users with minimal delay. AI algorithms update the IIFS database and make predictions of the interference. The interference reports will be available to all users to support their spectrum management objectives. This cyber physical system behavior in growth is expected to follow the pattern of database growth for WPS services [5]. Services providers can benefit from the IIFS database for interference management and ingrate it into the design of their cloud and edge computing facilities to optimize the delay in communications with the devices carrying multi-band chipsets and deliver the report to devices without multi-band receivers.

## Conclusions

This paper presented an overview of the issues regarding intelligent spectrum management and the role of interference monitoring and forecasting in the evolution of liberated spectrum wireless communications networks. We presented the views of different industries contributing to the RF cloud interference and spectrum management in licensed, unlicensed, and shared spectrum. We also provided examples of un-realistic interpretations of interference and how they caused delays in the growth of the wireless communications industry. Finally, we provided a model for the short time behavior of the interference using a circular scattering model for interference and proposed an architecture for an intelligent interference forecasting system based on models for evolution of Wi-Fi positioning systems.

## Footnotes

1 Typical distance-power gradient in urban areas in line-of-sight (LOS) communications is 2, and obstructed LOS it is 4. In indoors, LOS stays the same and in OLOS it is reduced to 3.5.

2 The legacy IEEE 802.11 took approximately ten years to finalize in 1997.

## References

[1] A. Viterbi, Spread spectrum communications--Myths and realities. *IEEE Communications Magazine*, *17*(3), pp. 11-18, 1979. (reprinted in May 2002 in 50th anniversary Commemorative Issue). (http://xanthippi.ceid.upatras.gr/courses/mobile/2004_05/SpreadSpectrum.pdf )

[2] Pahlavan K (1985). Wireless communications for office information networks. IEEE Communications Magazine.

[3] Pahlavan K, Ying J, Li Z, Solovey E, Loftus JP, Dong Z (2020). RF cloud for cyberspace intelligence. IEEE Access.

[4] P. Misra, A unified approach to consistent location determination, proccedings of the first rf localization for next generation wireless devices, Worcester Polytechnic Institute, Worcester, MA, 2008. http://www.cwins.wpi.edu/workshop08/program.html.

[5] Pahlavan K, Akgul F, Ye Y, Morgan T, Alizadeh-Shabdiz F, Heidari M, Steger C (2010). Taking positioning indoors Wi-Fi localization and GNSS. Inside GNSS.

[6] Pahlavan K (2019). Indoor Geolocation Science and Technology—At Emergence of Smart World and IoT.

[7] K. Pahlavan, P. Krishnamurthy, Evolution and impact of Wi-Fi technology and applications: a historical perspective. *International Journal of Wireless Information Networks*, pp. 1–17, 2021

[8] BEST-NEST Invitational Online Workshop on “New Paradigms in Intelligent Spectrum Management and Regulations, Future Directions, Technologies, Standards, and Applications. Dec 3-4, 2020, Webinar from WPI, Worcester, MA (https://bestnest.wpi.edu/index.php/synapsis-2/)

[9] K. Pahlavan, Understanding communications networks—for emerging cybernetics applications forthcoming, River Publishers, The Netherlands, 2021.

[10] M.I. Skolnik, *Radar handbook*. McGraw-Hill Education, 2008.

[11] Friis HT (1946). A note on a simple transmission formula. Proceedings of the IRE.

[12] Pahlavan K, Levesque AH (1995). Wireless information networks.

[13] B. Parkinson, Experiences, and Issues of. The US PNT (Position Navigation and Time). Advisory Board (PNTAB), Oct 22, 2019. Analogies to UAG of the National Space, https://rntfnd.org/wp-content/uploads/Brad-at-UAG-Users-Advisory-Group-Oct-2019.pdf.

[14] Marcus M (1987). Regulatory policy considerations for radio local area networks. IEEE Communications Magazine.

[15] S. J Shellhammer, A. K. Sadek, W. Zhang, Technical challenges for cognitive radio in the TV white space spectrum. In *2009 Information Theory and Applications Workshop* (pp. 323-333). IEEE, 2009.

[16] Wang J, Ghosh M, Challapali K (2011). Emerging cognitive radio applications: A survey. IEEE Communications Magazine.

[17] Hazlett TW (2017). The Political Spectrum: The Tumultuous Liberation of Wireless Technology, from Herbert Hoover to the Smartphone.

[18] K. Mun, CBRS: New shared spectrum enables flexible indoor and outdoor mobile solutions and new business models. *White Paper, Mar*, 2017.

[19] Lagunas E, Tsinos CG, Sharma SK, Chatzinotas S (2020). 5G cellular and fixed satellite service spectrum coexistence in C-band. IEEE Access.

[20] JUJITSU, High-Capacity Indoor Wireless Solutions: Picocell or Femtocell?, 2013. https://www.fujitsu.com/us/Images/High-Capacity-Indoor-Wireless.pdf

[21] T. Shields, Billionaires musk, ergen and dell brawling over spectrum at FCC, Bloomberg Wealth, 2021. https://www.bloomberg.com/news/articles/2021-10-09/billionaires-musk-ergen-and-dell-brawling-over-spectrum-at-fcc.

[22] J. Y. C. Cheah, Interference characteristics of microwave ovens in indoor radio communications. In *1*^*st*^* IEEE Workshop on Wireless Local Area Networks *(pp. 17-22), 1991.

[23] J.Y.C. Cheah, Interference characteristics of microwave ovens in indoor radio communications. In *PIMRC* (pp. 280-285), 1991.

[24] M. V. S. Chandrashekhar, P. Choi, K. Maver, R. Sieber, K. Pahlavan, Evaluation of interference between IEEE 802.11 b and Bluetooth in a typical office environment. In *12th IEEE International Symposium on Personal, Indoor and Mobile Radio Communications. PIMRC 2001. Proceedings (Cat. No. 01TH8598)*, vol. 1, pp. D-D. IEEE, 2001.

[25] Pahlavan K, Krishnamurthy P (2013). Principles of wireless access and localization.

[26] S. Cheng, K. Pahlavan, H. Wei, Z. Su, R. Zekavat, A. Abedi, Empirical interference analysis between mmWave Radars and IEEE 802.11AD, IJWIN, Springer Nature, (submitted for publication), 2021.

[27] IEEE 802.15.2-2003 - IEEE Recommended Practice for Information technology -- Local and metropolitan area networks-- Specific requirements -- Part 15.2: Coexistence of Wireless Personal Area Networks with Other Wireless Devices Operating in Unlicensed Frequency Bands. https://ieeexplore.ieee.org/stamp/stamp.jsp?tp=&arnumber=1237540

[28] Steve Jobs on Skyhook, Keynote at MacWorls, 2008. https://www.youtube.com/watch?v=r-xBFIApg2M.

[29] Pahlavan K, Krishnamurthy P, Geng Y (2015). Localization challenges for the emergence of the smart world. IEEE Access.

[30] Clarke RH (1968). A statistical theory of mobile-radio reception. Bell System Technical Journal.

[31] Zhang L, Liang YC, Xiao M (2018). Spectrum sharing for Internet of Things: A survey. IEEE Wireless Communications.

[32] K. Pahlavan, Intelligent server database for updating interference characteristics for spectrum sharing, US Patent Application 63/199,195, filed on 12/13/2020.

[33] R. K. Jones, F. Alizadeh-Shabdiz, E. J. Morgan, M. G. Shean, Skyhook Wireless Inc, 2008. *Server for updating location beacon database,* US Patent 7,414,988.

[34] E. J. Morgan, F. Alizadeh-Shabdiz, R. K. Jones, M. G. Shean, Skyhook Wireless Inc, 2008. *Method and system for building a location beacon database*. U.S. Patent 7,403,762.

[35] I. Goodfellow, J. Pouget-Abadie, M. Mirza, B. Xu, D. Warde-Farley, S. Ozair, A. Courville, Y. Bengio, Generative adversarial nets. *Advances in neural information processing systems*, 27, 2014.

[36] Jiang Q, Ma Y, Liu K, Dou Z (2016). A probabilistic radio map construction scheme for crowdsourcing-based fingerprinting localization. IEEE Sensors Journal.

